# Pictorial methods to assess heavy menstrual bleeding in research and clinical practice: a systematic literature review

**DOI:** 10.1186/s12905-020-0887-y

**Published:** 2020-02-10

**Authors:** Julia L. Magnay, Shaughn O’Brien, Christoph Gerlinger, Christian Seitz

**Affiliations:** 1 Institute for Science and Technology in Medicine, Guy Hilton Research Centre, Hartshill, Stoke-on-Trent, UK; 2 Department of Obstetrics & Gynaecology, Keele University School of Medicine, Stoke-on-Trent, UK; 3 Bayer AG, Building P300, 13342 Berlin, Germany; 4 Gynaecology, Obstetrics and Reproductive Medicine, University of Saarland Medical School, Homburg, Saar Germany

**Keywords:** Menstrual blood loss, Pictorial blood loss assessment chart, Alkaline hematin, Heavy menstrual bleeding, Menstrual pictogram, Abnormal uterine bleeding, Bleeding disorders, Uterine fibroids, Endometriosis

## Abstract

**Background:**

Pictorial blood loss assessment charts (PBACs) represent the most widely used method to assess menstrual blood loss (MBL) in clinical trials. The aims of this review were to: (1) determine the diagnostic accuracy of PBACs that have been validated against the reference alkaline hematin technique; (2) categorize the pitfalls of using obsolete and nonvalidated charts; (3) provide guidelines for development of a new PBAC or use of an existing chart to measure MBL in clinical trials; and (4) consider the feasibility of using pictorial charts in primary care.

**Methods:**

A literature review was conducted using Embase and MEDLINE databases. The review identified reports of women with self-perceived or actual heavy menstrual bleeding (HMB), bleeding disorders, abnormal uterine bleeding, leiomyomata (uterine fibroids) or endometriosis, and women undergoing treatment for HMB, as well as those with normal menstrual periods. Data were reviewed from studies that focused on the development and validation of PBACs and from those that used derivative noncertified charts to assess HMB.

**Results:**

Nine studies reported validation of PBAC scoring systems against the alkaline hematin technique. Across these studies, the sensitivity was 58–97%, the specificity was 7.5–95.5%, the positive and negative likelihood ratios were 1.1–13.8 and 0.14–0.56, respectively, and the diagnostic odds ratio was 2.6–52.4. The cut-off score above which the diagnosis of HMB was made ranged from 50 to 185. Several modifications of these PBACs were used in other studies; however, objective confirmation of their validity was not reported. Overall, there was widespread inconsistency of chart design, scoring systems, diagnostic cut-off limits and post-treatment outcome measures.

**Conclusions:**

PBACs are best suited to the controlled and specific environment of clinical studies, where clinical outcome parameters are defined. The current lack of standardization precludes widespread use of the PBAC in primary care.

**Review registration number:**

PROSPERO international prospective register of systematic reviews: CRD42016030083.

## Background

Heavy menstrual bleeding (HMB) is a common gynecological condition that adversely affects quality of life and work productivity. Approximately 10–35% of women report heavy menstrual periods at some stage during their reproductive years, with 5% consulting a medical practitioner for investigation of HMB [1]. In research, HMB is defined as a measured menstrual blood loss (MBL) of > 80 mL per cycle, but studies have repeatedly shown that at least 40% of women seeking medical attention for heavy periods lose less than this volume [2–5]. In clinical practice, the decision to treat is usually based on a woman’s self-reported symptoms and the effect of these symptoms on quality of life rather than any objective measurement, which concurs with current guidelines for management of HMB [6]. However, self-perception of menstrual loss is unreliable [4, 7].

Many clinical trial protocols require treatment efficacy for HMB to be determined by quantitative changes in MBL before a license can be granted for a new drug or surgical procedure. The perceived gold-standard method to measure MBL is the alkaline hematin (AH) technique, which was established in 1964, with later modifications [3, 8–12]. It is an expensive procedure that requires specialized laboratory facilities. Patients must collect, store and then submit all their used feminine products for MBL analysis, which may not be acceptable or feasible for many women. Therefore, it is mainly confined to clinical trials and the research setting to confirm or refute HMB and to evaluate efficacy of medical or surgical treatments.

An alternative semiquantitative method uses a pictorial blood loss assessment chart (PBAC) to assess MBL. This simple, inexpensive tool comprises a visual scoring system that depicts a graded series of soiled tampons and/or towels. The patient can directly record the number of her used feminine items and the degree to which they are bloodstained. Since its inception in 1990, the PBAC has become increasingly accepted by regulatory bodies as a substitute for the AH technique [13] and is now the most widely used method in clinical studies to confirm HMB and to measure response to treatment [14, 15]. It has also been used to measure postpartum blood loss [16, 17], to screen women for investigation of hemostatic disorders for which HMB may be a key symptom (e.g. von Willebrand disease) [18–20] and to evaluate the cost-effectiveness of different therapies for HMB [21, 22]. Two studies have used a pictorial chart to measure an increase in MBL after treatment of amenorrhea, or infrequent menstrual periods, with agents containing herbal preparations [23, 24].

To validate any method, it is crucial to have a standard against which the index test can be objectively judged. The AH technique is the most obvious comparator for the PBAC, but it only measures the blood fraction of menstrual discharge. Blood typically comprises approximately 50% of total menstrual volume, although this can vary widely on individual soiled sanitary items, particularly at extremes of menstrual loss [5, 25, 26]. However, feminine towels and tampons absorb all menstrual flow. To maintain parity with AH, validated PBAC icon scores represent blood loss, irrespective of the total fluid volume. In this review, we identify studies that describe the validation of key pictorial charts that have been used to measure MBL. We highlight the diversity in their diagnostic accuracy, the lack of standardization in their application and the pitfalls of using derivative noncertified PBACs. We propose essential criteria to bear in mind when using or validating a pictorial chart for study trials and consider the feasibility of their application in routine clinical practice.

## Methods

**Fig. 1:**
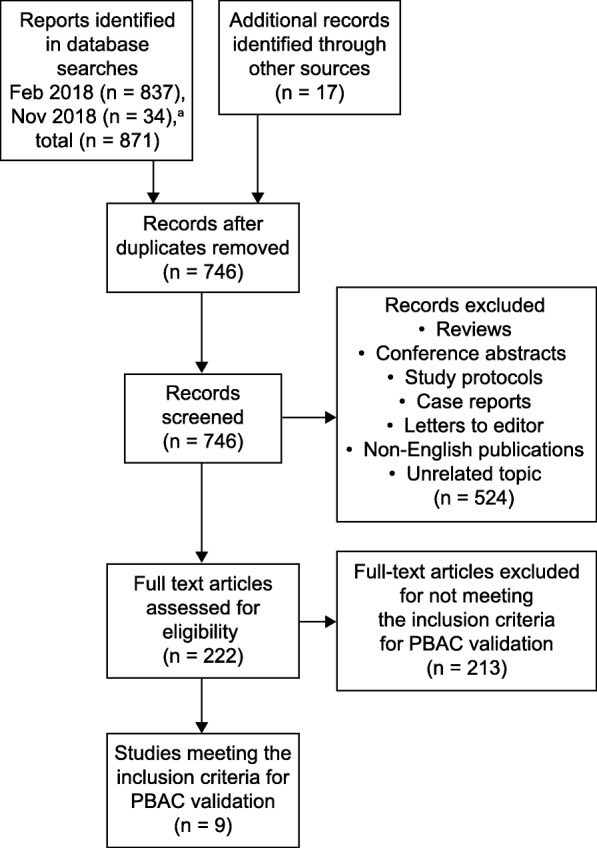
Database search and selection process. ^a^Additional references to those previously identified

Recommendations for the validation and use of PBACs were based on a systematic review of the electronic databases Embase and MEDLINE in February 2018, and again in November 2018. The review protocol was registered at PROSPERO (https://www.crd.york.ac.uk/PROSPERO) in March 2016: record number CRD42016030083. The search terms and selection process are shown in Additional file 1 and in Fig. 1. Articles were independently screened by two of the authors (J.L.M. and C.S.) for inclusion criteria. To be included in the literature review, articles were assessed for eligibility in terms of patients, outcomes and study design. The initial objective was to detect reports in which a pictorial chart had been used to measure MBL. The search was then specifically focused on studies that validated a PBAC against the AH method. The primary outcome was defined as the diagnostic accuracy of pictorial charts to determine HMB compared with objective quantitation; a volume of > 80 mL measured by the AH technique was considered to be diagnostic of excessive MBL [7]. The relevant data were extracted from text, tables or figures within the reports, and the quality of each study was evaluated using the QUADAS tool for the quality assessment of diagnostic accuracy studies; see Additional file 2 (Whiting et al. 2006 [27]). The diagnostic accuracy of each validated chart was calculated in terms of sensitivity, specificity, positive and negative likelihood ratios (LR + and LR−, respectively), and diagnostic odds ratio (DOR) [28]. In this setting, diagnostic accuracy was defined as the ability of a PBAC to discriminate between HMB and normal blood loss. Results that exceeded the stated cut-off threshold were classed as positive (HMB confirmed), and those below the threshold were classed as negative (HMB excluded).

## Results

From Embase and MEDLINE, 837 records were retrieved on February 11, 2018, and a further 34 were retrieved on November 2, 2018. After removal of duplicates and articles that did not validate a PBAC against the AH method, nine validation studies were identified. Reported quality parameters of the nine studies were generally satisfactory, and no studies were considered to have a very high risk of bias (Additional file 2). However, with one exception (Magnay et al. 2014 [5]), it was unclear whether PBAC test scores were interpreted without knowledge of AH results, and vice versa (*test review bias* and *reference review bias*, respectively). Also, it was unclear whether clinical information would normally be available when the index test is used in practice (*clinical review bias*). Statistical data for the individual studies are summarized in Table 1. The pooled population from these reports comprised 1347 women with a wide spectrum of MBL levels. This corresponded to 1821 menstrual cycles for which AH data and self-assessed PBAC scores were available. Across these nine studies, the diagnostic threshold score ranged from 50 to 185. The sensitivity was 58–97%, the specificity was 7.5–95.5%, the LR+ was 1.1–13.8 and the LR− was 0.14–0.56. The DOR was in the range 2.6–52.4, with the menstrual pictogram (superabsorbent-polymer-containing [SAP-c] version) giving the highest value [5]. It is generally accepted that the greater the DOR, the better the discriminatory test performance. However, measures of diagnostic accuracy are not fixed indicators of test performance. The diagnostic accuracy of one study may not apply to other patient groups and settings – a fact that is often overlooked. It was not feasible to pool data for meta-analysis because of the wide diversity in patient demographics, HMB prevalence, sanitary products, study procedures, cut-off scores and data analysis.

**Table 1:** Diagnostic accuracy of pictorial methods to determine heavy menstrual bleeding

Reference	N subjects (cycles)	Study population	HMB cut-off score	Sanitary products	Sensitivity, %	Specificity, %	LR+ ratio	LR− ratio	DOR
Higham et al. (1990) [29]	28 (55)	NR	100	Kotex Simplicity 2 Tampax/Kotex Fems Super Plus	86	89	7.8	0.16	49.7
Deeny et al. (1994) [30]	53 (53)	DUB	100	Not specified	88	52	1.8	0.23	7.9
Janssen et al. (1995) [31]	288 (489)	HMB or unexplained anemia	185	Kotex Maxi Long Tampax Super	62	95.5	13.8	0.40	34.6
Barr et al. (1999) [32]	281 (281)	Normal MBL	50	Not specified	58	75	2.3	0.56	4.1
Reid et al. (2000) [33]	103 (103)	Self-reported HMB	100	Kotex Simplicity 2 Tampax Super	97	7.5	1.1	0.40	2.6
Wyatt et al. (2001) [34]	108 (108)	Self-reported normal or HMB	80 mL	Kotex Maxi Day & Night Tampax Regular/Super/Super Plus	86	88	7.2	0.16	45.0
Zakherah et al. (2011) [35]	197(241)	Self-reported normal or HMB	150	Always Ultra No tampons	83	77	3.6	0.22	16.3
Larsen et al. (2013) [36]	170 (256)	UF with HMB	80 mL	Kotex Maxi Day & Night Tampax Regular/Super/Super Plus	88	87	6.8	0.14	49.1
Magnay et al. (2014) [5]	119 (235)	Self-reported light, normal or HMB	80 mL	Always Ultra Normal/Long/Night No tampons	82	92	10.3	0.20	52.4

*N* is the number of study subjects (number of menstrual cycles) for which data are available. *DOR* diagnostic odds ratio, *DUB* dysfunctional uterine bleeding, *HMB* heavy menstrual bleeding, LR likelihood ratio, *MBL* menstrual blood loss, NR not reported, UF uterine fibroids

The nine validation studies are discussed below in chronological order of publication, demonstrating the processes required to develop and to certify a pictorial method to measure MBL.

### Early validation studies

**Fig. 2:**
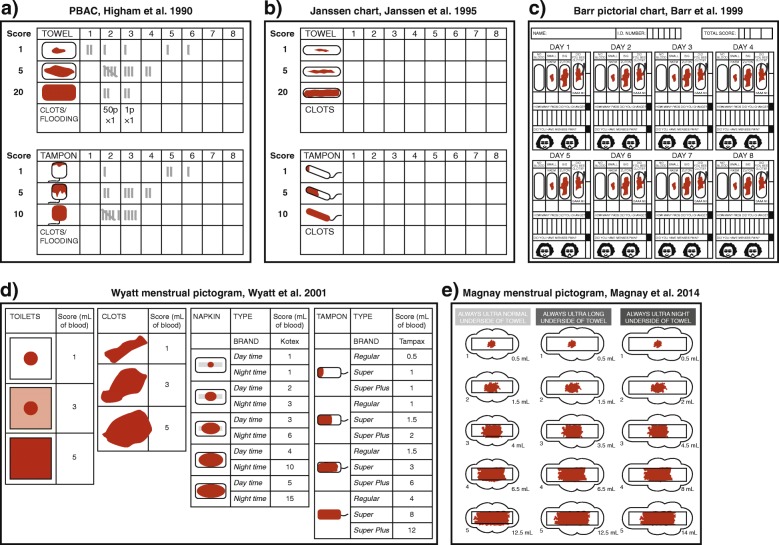
Examples of validated PBACs reported in the literature. *PBAC* pictorial blood loss assessment chart. **a**. The PBAC presented by Higham et al. reproduced from *British Journal of Obstetrics and Gynaecology*, 97, Higham JM et al. Assessment of menstrual blood loss using a pictorial chart, pp734–9, Copyright 2005, with permission from John Wiley and Sons [29]. **b**. The chart presented by Janssen et al. Adapted from *Obstetrics & Gynecology*, 85, Janssen CA et al. A simple visual assessment technique to discriminate between menorrhagia and normal menstrual blood loss, pp977–82, Copyright 1995, with permission from Elsevier [31]. **c**. The pictorial chart presented by Barr et al. Reproduced from *International Journal of Gynecology & Obstetrics*, 66, Barr F et al. A pictorial chart for managing common menstrual disorders in Nigerian adolescents, pp51–3, Copyright 1999, with permission from John Wiley and Sons [32]. **d**. The menstrual pictogram presented by Wyatt et al. Reprinted from *Fertility and Sterility*, 76, Wyatt KM et al., Determination of total menstrual blood loss, pp125–33, Copyright 2001, with permission from Elsevier; and adapted from *Obstetrician & Gynaecologist,* 6, Warrilow G et al. Quantification of menstrual blood loss, pp88–92, Copyright 2004 Royal College of Obstetricians and Gynaecologists, with permission from John Wiley and Sons [34, 37]. **e**. The chart presented by Magnay et al. Reprinted from *Fertility and Sterility,* 101, Magnay JL et al. Validation of a new menstrual pictogram (superabsorbent polymer-c version) for use with ultraslim towels that contain superabsorbent polymers, pp515–22, Copyright 2014, with permission from Elsevier [5]

The first PBAC was introduced in 1990 by Higham et al. and depicted three images (icons) that represented specific brands of feminine items soiled with increasing amounts of blood [29]. The icon scores were 1, 5 and 20 points for towels and 1, 5 and 10 points for tampons. A range of blood volumes produced visually similar stain sizes. Consequently, the score allocated to each icon was related, but not necessarily equal, to the applied blood volume in milliliters. The size of blood clots was compared with the diameter of UK coinage, and the number of flooding events was recorded, although there were no associated scores. Twenty-eight women of undisclosed menstrual status were monitored between one and 3 cycles each, giving 55 patient-assessed cycles in total. The PBAC scores ranged from 5 to 456 (median 121), whereas MBL measured by the AH technique ranged from 2 to 366 (median 74), giving a correlation coefficient (*r*) of 0.847. When used as a diagnostic test for HMB, a score of ≥100 gave a reported sensitivity and specificity of 86 and 89%, respectively. However, the PBAC progressively underestimated blood volume as menstrual loss increased, which demonstrated an important limitation of the method for women with HMB. The Higham PBAC is shown in Fig. 2a.

This PBAC was reassessed in 1994, although women were allowed to use their usual sanitary materials rather than those initially validated. With a specificity of 52%, it detected more false positives than the Higham study [30]. A year later, a modified pictorial chart was certified for use with specific feminine products. The scoring system was identical to the Higham PBAC, but the icons looked different (Fig. 2b). Based on receiver operating characteristic analysis, a cut-off point of 185 showed the greatest positive and negative predictive values for HMB, and it was concluded that a score of > 185, rather than > 100, should be used as a diagnostic threshold [31]. In 1999, adolescent Nigerian girls tested a novel pictorial chart with four towel icons (Fig. 2c). The sanitary products were not identified. Maximum sensitivity and specificity were achieved at a cut-off score of only 50, which was attributed to the fact that all the study population had normal MBL [32]. In 2000, the validity of the PBAC was challenged when Reid et al. tried to reproduce findings from the Higham pictorial chart on a population of women with self-reported HMB. Based on a specificity of 7.5% and a low correlation (*r* = 0.47) between PBAC score and AH, it was judged to be an inappropriate method to measure MBL [33].

In 2001, a new version of the PBAC was introduced by Wyatt et al. The *menstrual pictogram* depicted five icons representing blood loss on towels and four icons for tampons. The method was validated using simulated menstrual fluid (blood/saline, 1:1 ratio) to represent the physiological setting, in which the visible stain typically comprises about 50% blood (J.L. Magnay and K.M. Wyatt, personal communication). Scores for blood clots and extraneous loss were included. Crucially, all scores were quoted as blood volume with a cut-off limit of 80 mL, which made it directly comparable to the AH technique. The pictogram was validated using specified towel and tampon brands with various absorbency ratings (Fig. 2d) [34].

### Later validation studies

**Fig 3.**
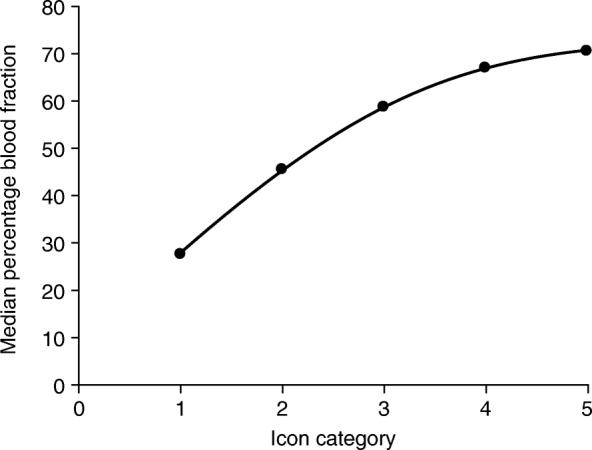
Median percentage blood fraction of menstrual fluid versus icon category of menstrual pictogram (SAP-c version). Second-order polynomial curve fit of median percentage blood fraction of menstrual discharge, with respect to icon category of the menstrual pictogram (SAP-c version); *n* = 3257 Always Ultra sanitary towels. *PBAC* pictorial blood loss assessment chart, *SAP-c* superabsorbent-polymer-containing. Reprinted from *Fertility and Sterility,* 101, Magnay JL et al., Validation of a new menstrual pictogram (superabsorbent polymer-c version) for use with ultraslim towels that contain superabsorbent polymers pp515–22, Copyright 2014, with permission from Elsevier [5].

Since these early validation studies, towel and tampon manufacturers have responded to market demand for more comfortable, discrete feminine products with enhanced fluid absorbency. Sanitary products used in the early PBACs became obsolete, and ultra-slim towels containing superabsorbent polymers rapidly gained popularity. In 2011, an updated version of the Higham chart was validated for Always Ultra towels (Proctor & Gamble, Cincinnati, OH, USA), the most popular brand of ultra-slim towel in the USA and UK [38, 39]. Tampons, clots and episodes of flooding were not evaluated so, to minimize extraneous loss, women were asked to use double towels during episodes of HMB. Maximum sensitivity and specificity were achieved at a cut-off score of 150 [35]. Two years later, Larsen et al. revalidated the menstrual pictogram after obtaining identical sanitary wear to that used in the original chart by Wyatt et al. (J.L. Magnay and L. Larsen, personal communication). At a threshold of 80 mL, the method gave a sensitivity of 96% and a specificity of 92% in dichotomizing response to medical treatment in terms of a ≥ 50% or < 50% decrease in MBL. This was the first time a pictorial chart had been endorsed for other than diagnostic purposes; namely, the MBL assessment of women with uterine fibroids treated with an investigational drug [36]. However, the validation was done with an obsolete version of feminine items and cannot be applied to other studies. In 2014, a new menstrual pictogram (SAP-c version) was validated with simulated menstrual fluid for use with Always Ultra towels and a choice of three absorbencies (Fig. 2e). Correction factors were applied to the provisional icon scores to account for the progressive increase in blood fraction with menstrual volume, as demonstrated in Fig. 3 [5, 26, 40]. The cut-off value for HMB was 80 mL.

### Nonvalidated PBACs

Several derivative PBACs were detected in our database search but, with the exception of those described above, objective confirmation of their validity was not reported. Most charts referenced the scoring system of Higham et al. and, to a lesser extent, Janssen et al., although several modifications were made. The brand(s) of sanitary products were stated for some PBACs [15, 41–45] but not for others [46–65]. The icon illustrations were often different and/or the individual scores were altered, whether by error or by design [41, 43, 46–49, 51, 52, 55–57, 60–63, 66, 67].

In numerous cases, a validated PBAC was referenced, but the cut-off threshold was adjusted for no obvious reason [46, 55–57, 59, 64, 65, 68–72]. Two reports directly compared the Higham and Janssen scoring systems (HMB > 100 and > 185, respectively) in the same study [73, 74]. In some instances, contemporary towels and tampons were deliberately substituted for their validated obsolete counterparts [16, 42, 75], but the cut-off score was not re-established. For a given volume, the visible stained area on modern sanitary materials is less than on older products [75] and can vary greatly between different brand formulations and absorbency ratings [41, 75–78]. Four studies directly converted PBAC score to MBL volume [17, 72, 79, 80]. However, this relationship is not linear because a range of blood volumes may be assigned to the same icon category. Three studies used a PBAC to simply assess the pattern of menstruation before and after treatment: the first to determine the length of menstrual period [81], the second to additionally record the number of towels used [82], and the third to measure the decrease in number of towels used at the time of heaviest menstrual loss [83]. None reported measured MBL. Table 2 lists some examples of modified, nonvalidated PBACs.

**Table 2:** Examples of nonvalidated PBACs to determine MBL

Publication	N	Sanitary products	Deviation from validated PBAC	Validated cut-off score for HMB	PBAC referenced
Biri et al. (2008) [46]	600	NR	• Icon-3 tampon score = 20 • Cut-off score = 50	100	Higham
Rott et al. (2009) [47]	46	NR	• Icon-3 tampon score = 15 • Icons different	100	Higham
Kouides et al. (2009) [42]	116	Kotex Curved Maxi Tampax Super	• Towel and tampon brands	100	Higham
Hacioglu et al. (2016) [61]	90	Free to choose	• Icons different	185	Janssen
Herman et al. (2016) [65]	900	NR	• Cut-off score = 150	100	Higham
Jacot-Guillarmod at el. (2010) [49]	N/A	NR	• Icon-3 tampon score = 20 • Icons different	100	Higham
Lopes et al. (2010) [41]	Lab tests	Kotex Ultraslim	• Icon-3 towel score = 10 • Icons different	100	Higham
Nahidi et al. (2011) [55]	160	NR	• Icons different • Cut-off score quoted as 80 mL	100	Higham
Donnez et al. (2015) [54]	242	‘Standardized’, brand not identified	• Clots equated to circle diameters	100	Higham
Dasharathy et al. (2012) [57]	201	Free to choose	• Number of towel icons = 4 • Icons different • Cut-off score = 72.5	80 mL	Wyatt
Hashim et al. (2012) [15]	95	Always Ultra Core Plus	• Towel brand	185	Janssen
Goshtasebi et al. (2013) [44]	90	Panberes	• Towel brand	185	Janssen
Hald et al. (2014) [56]	429	Free to choose	• Icons different • Cut-off score = 160	100	Higham
Mawet et al. (2014) [43]	280	Always Ultra Normal/Super Plus	• Towel brand • Icons different	80 mL	Wyatt
Brôlmann et al. (2016) [59]	50	NR	• Cut-off score = 120	80 mL	Wyatt
Ashraf et al. (2017) [60]	152	NR	• No stains on towel icons 1 and 3 or tampon icon 3	100	Higham
Han et al. (2018) [71]	95	NR	• Cut-off score = 130	185	Janssen
Gopimohan et al. (2015) [69]	45	Stayfree Secure Regular	• Cut-off score = 100	150	Zakherah
Gorgen et al. (2009) [64]	60	Unknown	• Cut-off score = 75	100	Higham
Barrington et al. (1997) [62]	50	‘Same brand used’ Not identified	• Icons different • Scoring system different	100	Higham
Van Dongen et al. (2009) [72]	21	NR	• Cut-off score = 200	100	Higham
Kashefi et al. (2015) [63]	71	‘Same towels used’ Not identified	• Icon-3 tampon score = 20	NR	Higham

*N* is the number of participants in study for which data are available. *HMB* heavy menstrual bleeding, *MBL* menstrual blood loss, *NR* not reported*, PBAC* pictorial blood loss assessment chart

## Discussion

Clinical trials often require a quantitative change in MBL as evidence of treatment efficacy for HMB. If a PBAC is used for this purpose, a decision must be made whether to use a validated method or to develop a new device. If the plan is to use an existing chart, attention should be focused on those certified for modern products [5, 35]. Authors should try to identify the study design that most closely matches their own setting. The prevalence of HMB in the reference PBAC report can be used as a guide if that of the intended study population is known [84].

With only two current charts to consider, the choice is limited. However, development of a new pictorial chart can significantly increase the cost and timeline of a clinical trial. The fundamental requirements for the design and validation of a PBAC are listed in Table 3 and should be carefully deliberated before a decision is made. Due consideration should be given whether to include scores for stained sanitary towels, stained tampons, menstrual clots and extraneous blood loss/flooding episodes. Many charts do not incorporate all these components, including some validated methods (Table 4). Other key factors are the construction and design, scoring system, ease and practicality of use and limitations of the method. Each of these elements is discussed below.

**Table 3:** Key requirements for the design and validation of PBACs

• Towel and tampon scores must be validated against the AH method • Scores must be validated for specific towel/tampon brands and for each absorbency category shown on the chart • To prevent mis-scoring or confusion, there must be clear visual differences between successive icons • To assess reproducibility during validation, icon scores should be subjected to repeated testing by different women • The chart must be validated for all levels of MBL (low, normal and high) • The method should be able to detect clinically relevant differences in MBL following treatment • The chart design should be simple, straightforward and user friendly, and patients should be thoroughly briefed in its use, particularly in the interpretation of nonstandard staining patterns • After validation, the format, size and appearance of the chart should not be changed because this may alter a woman’s perception of scoring • A paper-based version should be conveniently sized and of robust construction. If an electronic version is used, women should be aware of the protocol in cases of data transmission failure	

*AH* alkaline hematin, *MBL* menstrual blood loss, *PBAC* pictorial blood loss assessment chart

**Table 4:** Components included or excluded in validated pictorial methods to assess MBL

Reference	Component			
	Towels	Tampons	Clots	Flooding
Higham et al. (1990) [29]	✓	✓	✓	✓
Deeny et al. (1994) [30]	✓	✓	Not known	Not known
Janssen et al. (1995) [31]	✓	✓	✓	X
Barr et al. (1999) [32]	✓	X	✓	X
Reid et al. (2000) [33]	✓	✓	X	X
Wyatt et al. (2001) [34]	✓	✓	✓	✓
Zakherah et al. (2011) [35]	✓	X	X	X
Larsen et al. (2013) [36]	✓	✓	✓	X
Magnay et al. (2014) [5]	✓	X	X	X

*MBL* menstrual blood loss

### Components of a pictorial chart

#### Sanitary towels

**Fig. 4.**
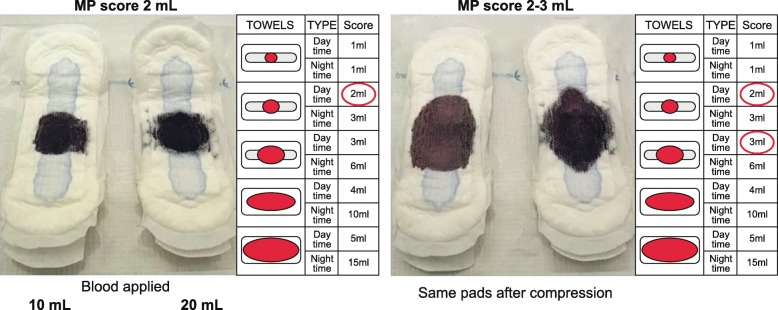
Measured blood volumes compared with the menstrual pictogram scores. Known volumes of blood were applied to Kotex Regular maxi towels to produce similar stain sizes to those depicted by the highlighted icon scores on the menstrual pictogram of Wyatt et al., which was validated using an older, obsolete version of the same sanitary product. The difference in volume required and the effect of towel compression on stain area are shown. *MP* menstrual pictogram. Reprinted from a poster with permission from the author: Burnett PE et al., Comparison of Menstrual Pictogram Scoring to the Validated Alkaline Hematin Assay as Techniques for Measuring Blood Loss on Feminine Hygiene Products [75]

All PBACs estimate MBL from feminine towels. Modern towels have a layered design, consisting of a fluid-permeable surface (topsheet), an absorbent core and an impermeable backing with adhesive. The shape, absorbency and size of the product may vary depending on the manufacturer. The towel contour may be either flat or curved, and with or without ‘wings’ which attach it securely in place and add extra leak protection. Menstrual fluid rapidly transfers to the absorbent core via a series of micro-funnels, leaving the upper surface relatively dry and stain free. The US Food and Drug Administration (FDA) regulates sanitary towels as Class I Medical Devices subject to manufacturing controls and consumer complaint management, but the absorbency ratings are arbitrary and unregulated. Towels of similar size and/or with the same stated absorbency category but from different manufacturers can have a wide range of fluid retention capacities [77]. In ultra-slim formulations, MBL may be captured by a thin collapsed polymeric foam layer that expands and absorbs fluid upon contact [85] or by superabsorbent polymer granules embedded in the towel core (e.g. Always Ultra). The manner of stain spread depends on the density and distribution of superabsorbent particles throughout the absorbent zone, which varies among towel brands. Modern thick maxi towels are more efficient at absorbing fluid and limiting stain spread than their former versions, as demonstrated in Fig. 4 [75], although they are generally less popular than ultra-slim products. A pictorial chart requires validation for a specific brand of feminine towel in a range of absorbencies to accommodate different levels of MBL.

#### Tampons

**Fig. 5:**
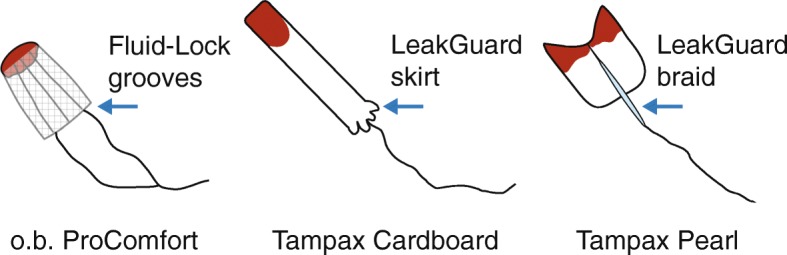
Examples of menstrual tampon shapes after addition of fluid. Anti-leak features of each tampon type are shown. When wet, o.b. ProComfort tampons swell radially to form a barrel shape, Tampax Cardboard tampons enlarge axially with minimal radial expansion and Tampax Pearl tampons expand to produce a winged profile. o.b. ProComfort tampons, Edgewell Personal Care, St Louis, MO, USA; Tampax tampons, Proctor & Gamble, Cincinnati, OH, USA

Tampon designs have progressively improved to limit leakage of menstrual fluid. Because they are worn internally, tampons are categorized by the US FDA as Class II Medical Devices. The maximum fluid retention weight for each absorbency category is strictly controlled, and all brands at the same rating must have the same maximum absorption capacity. Although most brands of unused tampons look essentially similar, the shape and dimensions of a worn item can vary considerably depending on the expansion and absorbency properties of the materials used. Figure 5 shows examples of tampon shapes after the addition of fluid. Using tampons during a menstrual period is less likely to result in extraneous fluid loss. Some women prefer the option and added sense of security of wearing a tampon and towel together because it gives greater personal choice; furthermore, this may potentially lead to an increased likelihood of patient recruitment and compliance in clinical studies [5, 86]. However, some women do not like tampons, particularly at times of heavy flow, and they are not included in all PBACs. If tampon icons are featured, they should represent the staining patterns produced by that particular brand.

#### Menstrual clots

**Fig. 6:**
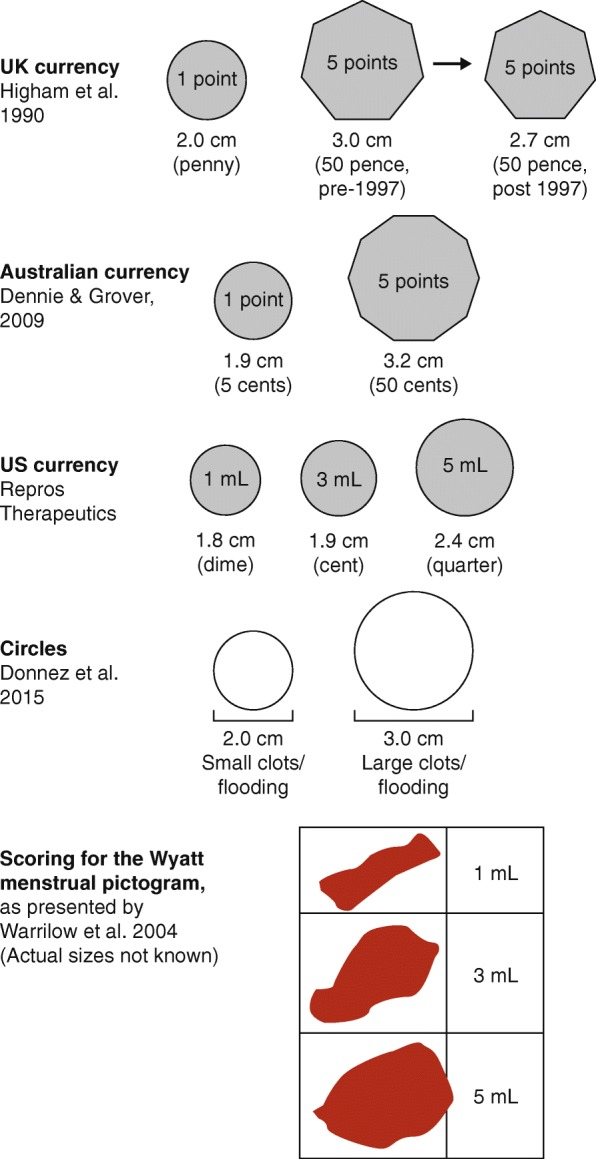
Examples of PBAC clot scoring icons. Actual-size diameters shown (cm) [29, 37, 52, 54, 88]. *PBAC* pictorial blood loss assessment chart. Clot scoring adapted from *Obstetrician & Gynaecologist*, 6, Warrilow G et al., Quantification of menstrual blood loss, pp88–92, Copyright 2004 Royal College of Obstetricians and Gynaecologists, with permission from John Wiley and Sons

The blood volume of menstrual clots is difficult to quantify. Clots can be quite gelatinous and contain varying amounts of endometrial tissue and vaginal secretions. The blood component cannot be verified by AH unless captured on sanitary wear and isolated for analysis. Many PBACs omit this element altogether [5, 33, 36, 47, 51, 56, 57, 87]. Some charts compare clot sizes to specific coinage [29, 31, 88] or to full-sized icons of stated diameters [54] (Fig. 6). The Wyatt pictogram depicts clots as a series of irregular shapes, but there is no associated size comparator [37]. Other PBACs use a subjective description of size – e.g. ‘small’ or ‘big/large’ – or just a ‘yes/no’ response, without reference to any measurable standard [32, 48, 49, 55]. Menstrual clot scoring is theoretically suitable for electronic versions of the PBAC if images are presented as actual-sized screenshots.

#### Extraneous blood loss (flooding episodes)

Extraneous blood loss is the passage of menstrual fluid that is not captured by a sanitary product. It can occur at times of excessive MBL, when the absorption capacity of the sanitary item is exceeded, or when there is nothing to absorb the menstrual flow, e.g. while bathing without a tampon inserted in the vagina. Opinions differ as to whether extraneous loss significantly contributes to the overall volume of menstrual discharge [7, 31, 32, 34], although it may be an important factor in influencing how women perceive their MBL. Noncaptured extraneous MBL is difficult to quantitate accurately because it cannot be measured by the AH technique. Most validated PBACs do not include flooding episodes (Table 4), nor do many derivative charts [47, 49, 51, 52, 56, 87]. Some require women to simply record the number of flooding events [41, 48] or to allocate 5 points for each flooding episode, regardless of severity [88, 89]. The Wyatt pictogram judges extraneous loss by scoring the graded decrease in visibility of a coin placed in the base of a toilet pan when incremental volumes of blood are added [34]. However, in the everyday setting, this measurement would depend on the dimensions of the toilet receptacle, the volume of diluting water and the availability of (nonretrievable) coins to perform the test.

### Scoring system

All included components of a PBAC should be assessed. The value assigned to individual towel and tampon icons should be verified against AH for every selected absorbency category. Allocating blood volumes to each icon (as opposed to an empirical value) allows direct assessment of MBL, as in the menstrual pictogram. Whichever scoring system is used, it must be sufficiently sensitive to detect what are considered to be clinically relevant differences in MBL at the appropriate time point following treatment for HMB. An important issue to consider is the increase in stain area when a feminine towel or tampon is subjected to mechanical load [75]. Compression forces exerted by the wearer may ultimately affect icon selection. Choosing products that show minimal stain spread under pressure would limit this confounding element [5, 86]. Other factors that can affect visible stain area are the rate and composition of menstrual flow, individual anatomy, physical activity and posture. It is difficult to control for these variables.

The PBAC must be tested by a spectrum of women with light, normal and heavy MBL to assess not only its validity as a diagnostic tool for HMB but also its ability to estimate normal and low menstrual volumes. Many women who complain of HMB actually have normal periods, and those with excessive blood loss often experience dramatic reductions in MBL after treatment. The scores should be reproducible and yield the same results in repeated applications [86].

### Construction and design

The format of a validated PBAC should not vary during use because it may change a patient’s perception of her MBL and, thus, her score. Because towels and tampons often have distinctive shapes and sizes, icon images need to represent the feminine items used [5, 86]. Successive icons must display clear differences in appearance to avoid indecision or mis-scoring. If a paper-based PBAC is chosen, the chart and menstrual diary should easily fit into a typical daytime-sized handbag and have a neutral, discrete cover. It must have a robust construction to prevent disintegration and allow sufficient room for the number of soiled feminine items to be recorded each day [86]. Prospective daily recording is critical for data collection involving a series of recurrent similar events such as MBL, in which the accuracy of retrospective recall tends to be low [90].

If an electronic design is preferred (e.g. a dedicated eDiary, smartphone application or website), a paper version may still be needed in situations in which telephone or Internet reception is unavailable, the equipment has insufficient battery charge, or it has been lost or damaged. Electronic recording of MBL is not a new concept. In 2002, a menstrual symptometrics device for use by patients incorporated a menstrual pictogram programmed into an Amstrad PDA600 PenPad [91]. It was validated for MBL measurement using the paper-based chart of Wyatt et al. as the reference standard [34]. Electronic data logging is generally preferred by patients and results in fewer missing data than a paper calendar and fewer transcription errors when results are transferred to a computer database [90]. A study showed that 80% of adolescents and young women preferred to use a smartphone application version of the PBAC over a paper-based chart, although the method was limited by mobile technology issues [92]. A second study developed an electronic PBAC (ePBAC) that could be completed using the Internet [93]. Neither chart had been validated against the AH method.

### Ease and practicality of use

To gain maximum patient compliance, use of the PBAC must be as straightforward as possible. At an early stage, patients’ views should be sought about the method and how it may be improved, and the level of compliance should be tested [86, 92]. Women should be supplied with sufficient feminine materials, based on their expected MBL, and carefully instructed on how to score their soiled items. This includes interpretation of nonstandard or fragmented staining patterns. The importance of prospective recording must be emphasized, and the contact details of a liaison nurse should be available in case of any concerns.

Extraneous MBL is common for women experiencing HMB and may be due to sudden flooding episodes, not changing sanitary products often enough or a combination of both. To limit this event, women should be encouraged to use tampons and towels concurrently, to ensure a fresh tampon is worn when showering/bathing or visiting the toilet and to change their feminine items as frequently as possible at times of heavy menstrual flow. If a woman does not wish to use tampons, she should choose towels with the highest absorbency rating and try to replace them before saturation occurs.

### Recognition of limitations

All diagnostic methods have limitations, particularly those with a subjective element. Each PBAC image is allocated a specific score, but patients may assign a range of blood volumes to the same icon category. The choice of icon may be influenced by whether a woman perceives her menstrual loss to be light, normal or excessive. A visual rating system is not as accurate as the AH method, which provides a definitive blood volume and does not rely on individual perception of MBL or errors in selecting or recording the correct icon. Pictorial charts may overestimate low volumes of MBL because whole blood represents a smaller proportion of the visible stain area than at normal and high volumes (Fig. 3). Conversely, the score assigned to the highest icon category can markedly underestimate MBL because of a ceiling effect as a sanitary item becomes fully saturated.

A potential drawback of using modern towels is the difficulty of visualizing the bloodstained area on the upper surface, because fluid is rapidly transferred to the interior region. The SAP-c menstrual pictogram overcomes this problem by scoring the stain on the underside of the towel, where it is clearly visible [5, 86]. Many stain profiles will not conform to the standardized PBAC icons [5, 94]. Stains may be fragmented or a completely different shape, which could potentially result in underestimation or overestimation of MBL, depending on personal perception of the soiled area.

Apart from the diversity in diagnostic accuracy – a fact highlighted by validated PBACs – there is a lack of standardization in measuring post-treatment outcomes. The aim of therapy is to improve a patient’s well-being to an acceptable level, but the relationship between quality of life and a clinically meaningful decrease in MBL remains unresolved. With a single exception, pictorial charts have not been validated as a tool to test the effectiveness of therapy [36], and researchers have had to define their own criteria for treatment success. Some have stipulated a decrease in PBAC score to below the chosen HMB cut-off value [95] or by a certain number of points from the baseline measurement of MBL, e.g. 50 points [15, 96–98]. Others have advocated a percentage reduction in score; values of 20–50% have been reported [36, 58, 59, 63, 99–105]. If the treatment goal is amenorrhea (PBAC score < 2), the endpoint is clearly defined [54, 106–109]. The aim of several reports is just to detect a significant difference in MBL reduction between different treatments for HMB.

The appropriate time point to test efficacy will need to be considered, subject to the type and purpose of therapy. Medical treatments designed to rapidly reduce MBL tend to have short follow-up times of between 1 month and 12 months [44, 110–113], whereas surgical interventions, such the levonorgestrel intrauterine system and endometrial ablation, are designed for long-term reduction of MBL. Monitoring of menstrual loss has been reported for up to 5 years after surgery at various time intervals, depending on the study [59, 106, 107, 114]. The post-treatment time point(s) for MBL measurement should be clearly defined in the trial design.

### Use of pictorial blood loss assessment charts in primary care

The role of PBACs in primary care is currently unclear. The usual procedure to assess MBL combines a woman’s self-perception of her menstrual flow (in terms of light, normal or heavy) with hemoglobin level and/or menstrual markers, such as length of period, number of used sanitary products, number of days of ‘heavy’ bleeding, number of flooding episodes and size of clots passed. Together with quality-of-life issues, this subjective approach is often the only trigger for medical and surgical interventions, although it is unreliable [2, 31, 76].

Measurement of MBL by the AH technique is obviously impractical in this setting, but a simple, semi-objective method would be a useful diagnostic tool. It could clarify the patient’s complaint and also influence the choice and expectations of treatment. Historical research data have shown that some women whose MBL has been shown to be normal can be dissuaded from seeking unnecessary therapy for perceived HMB [3, 115, 116]. A fully validated PBAC is theoretically suitable for this purpose, combined with a daily diary to document responses to specific health-related questions throughout the menstrual cycle. This day-to-day record would have the dual purpose of assessing both symptom severity and the impact of perceived or actual HMB on everyday life, which would comply with the National Institute for Health and Care Excellence (NICE) guidelines to assess quality-of-life issues for women with excessive blood loss. Although NICE guidelines do not recommend routine quantitation of MBL, they do acknowledge that further research into indirect measurements of MBL in primary and secondary care is warranted [6].

Some studies have recommended the use of a PBAC in primary care to increase the diagnostic accuracy of HMB [35, 36, 46], possibly as an adjunct to clinical history and menstrual markers [65, 117, 118]. However, widespread application of this method is currently limited by a lack of expert consensus regarding the choice of pictorial chart, the diagnostic cut-off score for HMB and clear objective criteria in the assessment of post-treatment clinical outcome.

At a practical level, the chart would require at least one menstrual cycle to complete. Results would not normally be available during a first consultation, unless a PBAC had been completed beforehand by prior arrangement with the healthcare center. A dedicated women’s clinic might suit this purpose, in which a nurse practitioner could train patients to use a validated PBAC, distribute the required sanitary protection and address any queries or anxieties [119]. Some women may be discouraged because of the inevitable delay in diagnosis, the rigorous attention to detail required when scoring MBL, the stipulation to change sanitary wear frequently to avoid leakage (which might not always be possible) or the need to use specified and possibly unfamiliar sanitary products. A diagnostic ‘primary care’ PBAC that has been validated for a range of popular sanitary products, particularly those designed for heavy menstrual flow, together with appropriate instruction and support may help to alleviate these concerns. Also, the widespread availability and popularity of smartphones opens the possibility of developing ePBACs that would be acceptable to patients. However, this area has yet to be explored.

## Conclusions

Pictorial blood loss assessment charts are increasingly being used in clinical trials to diagnose HMB and to evaluate efficacy of new treatments, despite the fact that, in most cases, their accuracy has not been validated with modern sanitary materials. We have highlighted some key factors to consider when using this method in the contemporary research setting. We hope that this review will act as a basic guide for researchers who wish to use a PBAC to assess MBL, whether for selection of the most appropriate validated method or the creation of a new chart tailored to the requirements of their study. Feminine hygiene technology will continue to advance, and the market should be constantly monitored for product updates. New devices for electronic data capture will also be developed and may become the method of choice for recording patient results. Pictorial charts must keep pace with these changes and be revalidated accordingly if they are to remain the chosen tool to assess MBL in clinical research. With these caveats in mind, we suggest that the PBAC can be a useful tool to determine MBL under defined and controlled study conditions.

## Supplementary information

**Additional file 1.** Electronic search strategy of Medline database, performed in NCBI Pubmed.

**Additional file 2.** The QUADAS questionnaire for the quality assessment of diagnostic accuracy studies.

**Additional file 3.** Completed Preferred Reporting Items for Systematic reviews and Meta-Analyses (PRISMA) Checklist

## Data Availability

A completed PRISMA checklist for the manuscript is provided as Additional file 3. Please note that the page numbers herein refer to the original submission file and not the final print version.

## Abbreviations

AH: alkaline hematin
DOR: diagnostic odds ratio
ePBAC: electronic pictorial blood loss assessment chart
FDA: Food and Drug Administration
HMB: heavy menstrual bleeding
LR+: positive likelihood ratio.
LR–: negative likelihood ratio
MBL: menstrual blood loss
NICE: National Institute for Health and Care Excellence
PBAC: pictorial blood loss assessment chart
SAP-c: superabsorbent-polymer-containing
