# Supplementary material for: Pictorial methods to assess heavy menstrual bleeding in research and clinical practice: a systematic literature review
Source: BMC Womens Health. 2020 Feb 10;20:24. doi: 10.1186/s12905-020-0887-y (PMC7011238; doi:10.1186/s12905-020-0887-y)
Supplement: Supplementary file 1 — Additional file 1. Electronic search strategy of Medline database, performed in NCBI Pubmed. [file 12905_2020_887_MOESM1_ESM.pdf]

Additional file 1.

Electronic search strategy of Medline database, performed in NCBI Pubmed 12 Feb 2018

Data coverage: no time limits imposed, search strategy: best match

USER QUERY

(((((((((menorrhagia) OR heavy menstrual bleeding) OR menstrual blood loss) OR abnormal uterine bleeding) OR bleeding disorders) OR uterine fibroids) OR leiomyomata) OR endometriosis)) AND alkaline hematin) OR ((((pictorial blood loss assessment chart) OR pictorial chart) OR pbac) OR menstrual pictogram)

1. Menorrhagia
2. Heavy menstrual bleeding
3. Menstrual blood loss
4. Abnormal uterine bleeding
5. Bleeding disorders
6. Uterine fibroids
7. Leiomyomata
8. Endometriosis
9. 1 OR 2 OR 3 OR 4 OR 5 OR 6 OR 7 OR 8
10. Alkaline hematin
11. Pictorial blood loss assessment chart
12. Pictorial chart
13. PBAC
14. Menstrual pictogram
15. 11 OR 12 OR 13 OR 14
16. 9 AND 10 OR 15

SEARCH INFORMATION

Medical Subheadings (MeSH) used

leiomyoma; menstruation; menorrhagia; blood coagulation disorders; hemorrhage; hemin; endometriosis; uterine hemorrhage; assessment; chart

Additional terms included

heavy menstrual bleeding; assessment; alkaline; pictorial; menstruates; losses; bleeding; blood; hemin; abnormal; menstruated; uterin; fibroids; coagulation; hemine; loss; blood coagulation disorders; uterine fibroids; haematoma; menstrually; blood loss; uterine; pictorials; menorrhagias; pictorially; haemin; bloods; disorder; uterine hemorrhage; leiomyomas; haemorrhage; coagulators; coagulational; charts; endometriosis; haematin; haemorrhagic; fibroid; menstruate; menstruations; hematin; pbac; leiomyomata; hemorrhaged; leiomyoma; heavy; assessments; panhematin; alkalin; haematology; disorders; uterine bleeding; bleeding disorders; hemorrhage; menstrual; hemorrhagically; bleedings; hemins; coagulations; menstruation; menstruating; menorrhagia; alkalines; pictograms; chart; pictogram; hemorrhages
