# Supplementary material for: Pictorial methods to assess heavy menstrual bleeding in research and clinical practice: a systematic literature review
Source: BMC Womens Health. 2020 Feb 10;20:24. doi: 10.1186/s12905-020-0887-y (PMC7011238; doi:10.1186/s12905-020-0887-y)
Supplement: Supplementary file 2 — Additional file 2. The QUADAS questionnaire for the quality assessment of diagnostic accuracy studies. [file 12905_2020_887_MOESM2_ESM.pdf]

Additional file 2. The QUADAS questionnaire for the quality assessment of diagnostic accuracy studies

Item *(characteristic tested)*	Higham [29]	Deeny [30]	Janssen [31]	Barr [32]	Reid [33]	Wyatt [34]	Zakherah [37]	Larsen [36]	Magnay [5]
1. Representative spectrum of *patients (spectrum composition)*	X	√	√	X	X	√	√	√	√
2. Clear selection criteria described *(selection criteria)*	√	√	√	√	√	√	√	√	√
3. Correct diagnostic classification by reference test *(reference standard)*	√	√	√	√	√	√	√	√	√
4. Short enough interval between index & reference test *(disease progression bias)*	√	√	√	√	√	√	√	√	√
5. Verification of diagnosis using reference test (*partial verification)*	√	√	√	√	√	√	√	√	√
6. Same reference test used for all patients *(differential verification)*	√	√	√	√	√	√	√	√	√
7. Independent index & reference tests *(incorporation bias)*	√	√	√	√	√	√	√	√	√
8. Sufficient description of index test *(index test execution)*	√	√	√	X	√	√	√	√	√
9. Sufficient description of reference test *(reference standard execution)*	√	√	√	√	X	√	X	X	√
10. Index test results without knowledge of reference results *(test review bias)*	U	U	U	U	U	U	U	U	√
11. Reference test results without knowledge of index test results *(reference review bias)*	U	U	U	U	U	U	U	U	√
12. Same clinical data available before interpretation of both index and reference tests *(clinical review bias)*	U	U	U	U	U	U	U	U	√
13. Uninterpretable or intermediate test results reported *(uninterpretable test results)*	N/A	N/A	N/A	N/A	√	N/A	N/A	N/A	N/A

14. Withdrawals from study explained *(withdrawals)*	√	N/A	√	√	N/A	√	√	N/A	√

Questionnaire adapted from Whiting et al (2006). A tick (√) indicates "yes", X indicates "no", "U" indicates "unclear" and N/A indicates "not applicable to study". Whiting PF, Weswood ME, Rutjes AW, Reitsma JB, Bossuyt PN, Kleijnen J. Evaluation of QUADAS, a tool for the quality assessment of diagnostic accuracy studies. BMC Medical Research Methodology 2006;6:9
